# Android Fat Deposition and Its Association With Cardiovascular Risk Factors in Overweight Young Males

**DOI:** 10.3389/fphys.2019.01162

**Published:** 2019-09-18

**Authors:** Carolina Ika Sari, Nina Eikelis, Geoffrey A. Head, Markus Schlaich, Peter Meikle, Gavin Lambert, Elisabeth Lambert

**Affiliations:** ^1^Human Neurotransmitters Laboratory, Baker Heart and Diabetes Institute, Melbourne, VIC, Australia; ^2^Iverson Health Innovation Research Institute, School of Health Sciences, Faculty of Health, Arts and Design, Swinburne University of Technology, Hawthorn, VIC, Australia; ^3^Neuropharmacology Laboratory, Baker Heart and Diabetes Institute, Melbourne, VIC, Australia; ^4^Dobney Hypertension Centre, School of Medicine – Royal Perth Hospital Unit, The University of Western Australia, Perth, WA, Australia; ^5^Metabolomics Laboratories, Baker Heart and Diabetes Institute, Melbourne, VIC, Australia

**Keywords:** overweight, android fat, endothelial function, cardiovascular risk, sympathetic activity

## Abstract

**Objective:**

Excess adiposity increases the risk of type-2 diabetes and cardiovascular disease development. Beyond the simple level of adiposity, the pattern of fat distribution may influence these risks. We sought to examine if higher android fat distribution was associated with different hemodynamic, metabolic or vascular profile compared to a lower accumulation of android fat deposits in young overweight males.

**Methods:**

Forty-six participants underwent dual-energy X-ray absorptiometry and were stratified into two groups. Group 1: low level of android fat (<9.5%) and group 2: high level of android fat (>9.5%). Assessments comprised measures of plasma lipid and glucose profile, blood pressure, endothelial function [reactive hyperemia index (RHI)] and muscle sympathetic nerve activity (MSNA).

**Results:**

There were no differences in weight, BMI, total body fat and lean mass between the two groups. Glucose tolerance and insulin resistance (fasting plasma insulin) were impaired in group 2 (*p* < 0.05). Levels of plasma triglycerides and 5 lipid species were higher in group 2 (*p* < 0.05). Endothelial function was less in group 2 (RHI: 1.64 vs. 2.26, *p* = 0.003) and heart rate was higher (76 vs. 67 bpm, *p* = 0.004). No difference occurred in MSNA nor blood pressure between the 2 groups.

**Conclusion:**

Preferential fat accumulation in the android compartment is associated with increased cardiovascular and metabolic risk via alteration of endothelial function.

## Introduction

Excess adiposity has in general been associated with both increased cardiovascular (CV) disease and all-cause mortality (Calle et al., 1999). Nonetheless, the link between obesity and mortality has recently been disputed (Vecchie et al., 2018). Body mass index (BMI), the most widely used measure of adiposity, may not be the most reliable tool to predict CV and metabolic risk because it does not differentiate between fat and lean mass or give an indication of fat distribution, i.e., visceral vs subcutaneous (Abramowitz et al., 2018). Many studies have demonstrated that excessive truncal or android fat (abdominal or visceral fat) may be the driving force behind increased CV disease development and progression to type-2 diabetes (Wiklund et al., 2008).

Increased android fat has been shown to be more closely associated with a clustering of metabolic syndrome components compared to gynoid fat in elderly people (Kang et al., 2011). Android fat is strongly correlated with serum lipids in population studies (Min and Min, 2015) and is associated with insulin resistance and diabetes in aging adults (Peterson et al., 2015). On the other hand, accumulation of fat in the lower body (gluteofemoral or gynoid regions) is associated with a more favorable lipid (Min and Min, 2015) and glucose profile as well as a decrease in CV and metabolic disease prevalence after adjustment for total body mass (Snijder et al., 2004).

Studies in younger populations have also demonstrated that android fat was more closely related to metabolic risk factors. For instance, the android/gynoid ratio was the obesity measure most closely related to both insulin resistance and dyslipidemia in children 7–13 years old (Samsell et al., 2014) and intra-abdominal fat was the most important component of the body fat for multiple metabolic risk factors in a group of young adults (von Eyben et al., 2003).

In addition to the metabolic consequences accompanying excess adiposity, we showed that being overweight was associated with decreased endothelial, renal and cardiac function suggestive of early markers of CV risk in young healthy adults (Lambert et al., 2010). Whether overweight-induced early organ damage is more related to android fat is unsure because this issue has not been investigated in detail. In middle aged subjects, android fat was found to be a determinant of arterial stiffness independent of traditional risk factors (Corrigan et al., 2017) and in a large study of subjects drawn from the general population, the trunk/body fat mass ratio was a predictor of early decline in kidney function (Oh et al., 2017). However, these studies included mostly lean participants, hence it remains uncertain as to whether early organ damage are more related to the presence of android fat in the overweight/obese setting.

Morphological and functional heterogeneity among adipose depots, together with genetic and environmental factors may contribute to differential cardiometabolic risk (Guglielmi and Sbraccia, 2018). Of note is the fact that sympathetic overdrive (Lambert et al., 2010) and elevated concentration of serum uric acid (UA) (Lambert et al., 2017a) are important drivers of early CV risk indices in overweight subjects. Sympathovagal imbalance in the form of sympathetic overactivity and/or vagal withdrawal has been recognized as the central pathophysiological mechanism involved in the genesis of obesity. Sympathovagal imbalance has been reported to be the potential contributor to the obesity related co-morbidities such as diabetes, insulin resistance, hypertension, dyslipidemia and CV dysfunctions (Indumathy et al., 2015). Sympathetic nervous system overactivity is likely to negatively impact on glucose metabolism, lipid profile, blood pressure and end organ damage (Lambert et al., 2010; Eikelis et al., 2017). Alvarez et al. (2002, 2004) showed that for the same level of BMI and total fat mass, subjects with high abdominal visceral fat have higher muscle sympathetic nerve activity (MSNA) compared to those with lower abdominal fat mass, while subcutaneous obesity was not associated with elevated sympathetic tone. Such sympathetic activation occurring preferentially in relation to the abdominal fat level may be an important link between abdominal obesity and the development of CV risk although this remains to be investigated. Serum UA has recently emerged as an important independent risk factor for increased CV disease (Borghi et al., 2018) and was found to be associated with endothelial dysfunction, arterial stiffness and decreased renal function in individuals free of CV disease (Lambert et al., 2017a). Some studies have suggested that increased serum UA may be more pronounced in subjects with increased visceral adiposity (Kim et al., 2012; Zhang et al., 2018) which may impact the CV risk profile.

Whether fat distribution is an important determinant of CV risk in young healthy overweight individuals and whether this is associated with autonomic nervous activity (sympathetic and vagal function) and serum UA remains uncertain. We hence evaluated the metabolic profile, end organ damage (renal, endothelial function and augmentation index), sympathetic nerve activity and serum UA concentration in healthy overweight men with low and high level of android fat.

## Materials and Methods

### Subject Selection

The current study subjects (*n* = 46) participated in a previous clinical investigation (Lambert et al., 2017b). They were all male and were recruited through two major universities in the Melbourne metropolitan area. Participants fulfilled the following criteria: BMI ≥25 kg/m^2^ and aged between 18 and 30 years. They were non- smokers and not on any medication. None of the participants had a history of CV, metabolic or cerebrovascular disease. The Alfred Hospital Human Ethics Committee approved the study protocol and all subjects gave written informed consent before participating in the study.

### Clinical Assessment

Participants were studied in the morning after an overnight fast. There were allowed one drink of water in the morning.

Demographic details of age, gender, race, clinical status and blood pressure were obtained from standard measurements and questionnaires. A detailed history and physical examination were conducted to exclude obesity and CV related comorbidities. Supine blood pressure was measured 3 times after 5 minutes rest using a Dinamap monitor (Model 1846SX, Critikon Inc., Tampa, FL, United States) and values were averaged. Body weight was measured in light indoor clothes without shoes using a digital scale. Waist circumference was measured at the midpoint between the lowest rib and iliac crest, and hip circumference at the level of the greater trochanters.

### Endothelial Function and Augmentation Index

The endothelial function was assessed using the digital pulse amplitude measured in the fasting state with a pulse amplitude tonometry (PAT) device placed on the tip of each index finger (Itamar Medical Ltd.). PAT was assessed in response to reactive hyperemia. Measurements were obtained for 5 to 10 min at baseline followed by 5 min of occlusion of 1 arm, with the cuff inflated on the upper arm to suprasystolic pressure (60 mm Hg above systolic pressure or 200 mm Hg) and then released to induce reactive flow-mediated hyperemia, measured for 5 to 10 min. The PAT ratio was calculated as $\log_{e}\left[\frac{X_{h\,t}}{X_{h\,0}}/\frac{X_{c\,t}}{X_{c\,0}}\right]$, with “X” representing pulse amplitude, “h” denoting hyperemic finger, “c” denoting the control finger, “t” denoting the 30-s time interval between 1.5 min and 2.0 min post deflation, and “0” denoting baseline. This calculation was made independent of the automatic algorithm provided by Itamar Medical Ltd., and was implemented in endothelial function assessment in the Framingham Heart Study (Hamburg et al., 2008). The pulse amplitude waveform analysis of the PAT signal was used to derive a measure of arterial stiffness and was expressed as augmentation index (AI) normalized to a heart rate of 75 bpm (AI@75).

### Muscle Sympathetic Nerve Activity, Heart Rate, and Blood Pressure

Recording of multiunit postganglionic MSNA was made with participants resting in a supine position. A tungsten microelectrode (FHC, Bowdoin, ME, United States) was inserted directly into the right peroneal nerve just below the fibular head. A subcutaneous reference electrode was positioned 2–3 cm away from the recording site. The nerve signal was amplified (350,000), filtered (bandpass 700–2000 Hz), rectified and integrated. During MSNA recording, blood pressure was measured continuously using the Finometer system (Finapress Medical System BV, Enschede, Netherlands), and heart rate was determine using a three-lead echocardiogram. Blood pressure, electrocardiogram data, and MSNA were digitized with a sampling frequency of 1000 Hz (PowerLab recording system, model ML 785/8SP; ADI Instruments, Bella Vista, Australia). Resting measurements were recorded over a 15-min period and averaged. The MSNA was expressed as burst frequency (burst/min) and burst incidence (bursts/100 heartbeats). In addition, all of the participants underwent ambulatory BP monitoring over 24–26 h using an oscillometric monitor (model No. 90207, SpaceLabs Medical Inc., Snoqualmie, WA, United States) to measure brachial blood pressure and heart rate every 30 min. Blood pressure and heart rate values were averaged over the total period of the recording.

### Spontaneous Cardiac Baroreflex Sensitivity

Baroreflex sensitivity was assessed using the sequence method (Parati et al., 1997). The baroreflex efficacy index (BEI) and slope of the regression line between cardiac interval and systolic blood pressure was calculated for each validated sequence and averaged during a 15-min supine recording.

### Heart Rate Variability

Heart rate variability (HRV) was assessed from the resting ECG recordings obtained during the MSNA recording and was determined using commercially available software (HRV Module for Chart 5 Pro; ADI Instruments, Bella Vista, Australia). Parameters derived were root mean square of successive R-R intervals (RMSSD) in the time domain analysis and Low Frequency (LF: 0.04–0.15 Hz) and High Frequency (HF: 0.15–0.4 Hz) in the frequency domain analysis expressed as normalized units.

### Biochemistry and Metabolic Measurements

Fasting blood samples were drawn from a cannula placed in an antecubital vein for biochemical analysis of creatinine, electrolytes, non-esterified fatty acids (NEFA), insulin, leptin, uric acid (UA), total cholesterol, triglycerides (TG), high-density lipoprotein (HDL), low-density lipoprotein (LDL), cholesterol, glucose, and liver enzymes alanine aminotransferase (ALT) and gamma-glutamyl transpeptidase (GGT). A standard 75-g oral glucose tolerance test (OGTT) was performed and another blood sample was withdrawn 120 min post glucose administration (Glucaid, Fronine PTY, LTD., Taren Point Australia). Fasting insulin levels was measured as a surrogate index for insulin resistance as this has been shown to a reliable measure in healthy subjects (Laakso, 1993).

The creatinine clearance was used to assess renal function. All the participants provided a 24-h urine collection on the day of the test. Creatinine clearance (*CCr*) was calculated using the following formula: *CCr*=(*U*_*Cr*_×*V*)/(*P*_*Cr*_), where “*U*_*Cr*_” is the creatinine concentration in urine, “*V*” the urine flow rate, and “*P*_*Cr*_” the creatinine concentration in plasma.

### Lipidomic Analysis

Lipidomic analysis was performed by liquid chromatography, electrospray ionization-tandem mass spectrometry using an Agilent 1290 liquid chromatography system with a 50x-mm Zorbax Eclipse Plus 1.8-mm C18 column combined with an Agilent 6490 mass spectrometer. The methods and lipid species within classes and subclasses analyzed have been described previously (Weir et al., 2013; Eikelis et al., 2017).

### Body Composition

Dual-energy X-ray absorptiometry scans were performed using Lunar iDXA (GE Health). Participants were wearing standard hospital gown. All jewelries were removed prior to the scan. The participants were lying down with their body fitted in the box outline on the iDXA table. All iDXA users were trained by the company with regards to correct placement. The iDXA unit was calibrated daily using the GE Health Lunar calibration phantom. Using this system, regional body composition precision error was previously reported to be less than 2.5% coefficient of variation for all regions except arms (Rothney et al., 2012).

Total body, android and gynoid fat and lean masses were determined using the software provided by the manufacturer. The GE Healthcare systems define the android region as the area between the ribs and the pelvis that is totally enclosed by the trunk region. The gynoid region includes the hips and upper thighs and overlaps both the leg and trunk regions (Imboden et al., 2017).

### Data Analysis and Statistics

The participants were divided into 2 groups (*n* = 23 each) according to the median value of the ratio of android fat to total body fat (%). Those above the median value (9.5%) were identified as “higher android fat content” and those below the value defined as “lower android fat content.” Linear regression analysis was performed to assess the difference between the 2 groups of subjects. The model included the 2 quantiles of the ratio and was adjusted for BMI. We assessed the validity of the models by plotting the residuals against quantiles of the normal distribution.

All statistical analyses were performed using Stata 14.0 (StataCorp, 2015. College Station, TX, United States).

## Results

The characteristics of the subjects are presented in Table 1. There was no difference in age and ethnicity between the 2 groups. Except for android fat mass, there was no significant difference in any other anthropometric measures between the 2 groups of subjects.

**TABLE 1:** Age, ethnicity and body composition.

	**Lower android fat content (<9.5% of total fat), *n* = 23**	**Higher android fat content (>9.5% of total fat), *n* = 23**	***P*-value**
Age, years	23 ± 3	23 ± 3	0.799
Ethnicity Asian/Caucasian (n)^(1)^	11/12	14/9	0.553
Body mass, kg^(2)^	94.0 (11.2)	97.8 (33.5)	0.517
BMI, kg/m^2(2)^	29.3 (3.8)	30.7 (7.9)	0.215
Waist circumference, cm^(2)^	96.5 (13.5)	105 (20.0)	0.118
Body fat,%	32.4 ± 6.9	35.7 ± 7.4	0.150
Total fat mass, kg^(2)^	29.4 (14.2)	36.3 (18.1)	0.103
Total lean mass, kg	60.8 ± 8.4	60.8 ± 10.2	0.981
Android fat mass, kg^(2)^	2.4 (1.4)	3.6 (2.1)	0.005
Gynoid fat mass, kg^(2)^	5.1 (1.5)	4.9 (3.9)	0.733

*Data is reported as mean ± SD or median (interquartile range). ^(1)^Chi-square with Yates correction, ^(2)^Mann–Whitney test.*

### Hemodynamic Assessments

Hemodynamic assessments are presented in Table 2. Systolic blood pressure and diastolic blood pressure as assessed either in the clinic or over a 24h period did not differ, but the heart rate was significantly higher in those with higher android fat content. Muscle sympathetic nerve activity (successful recordings in 45 subjects) as expressed in burst frequency was slightly higher in subjects with higher android fat (*p* = 0.04) but this significance was lost after adjustment for the heart rate (burst incidence, *p* = 0.68). Similarly, the slope and the BEI derived from the cardiac baroreflex function analysis were not different. None of the HRV parameters differed between the two groups.

**TABLE 2:** Blood pressure, heart rate, muscle sympathetic nerve activity (MSNA), and cardiac baroreflex function.

	**Lower android fat content (<9.5% of total fat)**	**Higher android fat content (>9.5% of total fat)**	***P*-value**
24-h systolic blood pressure, mmHg	120.0 ± 8.7	117.4 ± 8.2	0.324
24-h diastolic blood pressure, mmHg	70.0 ± 7.0	71.4 ± 8.7	0.554
24-h heart rate, bpm^(1)^	67.5 (8.8)	72.0 (8.0)	0.048
Clinic systolic blood pressure, mmHg	121.8 ± 12.6	120.6 ± 12.3	0.49
Clinic diastolic blood pressure, mmHg	69.7 ± 6.3	73.0 ± 8.9	0.34
Heart rate, bpm	67.2 ± 8.7	76.2 ± 10.9	0.01
MSNA, bursts per min	28.7 ± 11.6	31 ± 13.4	0.04
MSNA, bursts per 100 heartbeats	43.9 ± 16.0	44.3 ± 15.6	0.68
Cardiac baroreflex function slope, ms/mmHg	31.2 ± 15.4	29.7 ± 20.4	0.766
BEI	36.2 ± 13.9	32.4 ± 11.8	0.59
Heart rate variability components			
RMSSD, ms^(1)^	39.9 (25.9)	40.7 (25.9)	0.982
LF (nu)	61.3 ± 16.9	61.3 ± 12.0	0.989
HF (nu)	38.2 ± 16.1	37.3 ± 11.2	0.817
LF:HF^(1)^	1.60 (1.29)	1.82 (1.20)	0.590

*BEI, baroreflex efficacy index; RMSSD, root mean square of successive RR interval differences; LF, low frequency; HF, high frequency; nu, normalized units. Data is reported as mean ± SD or median (interquartile range). ^(1)^Mann–Whitney test.*

### Metabolic Parameters

The fasting plasma glucose concentration was not different between subjects with lower and higher android fat content, however, 2-h plasma glucose concentration as well as fasting insulin concentration were higher in those with more android fat content (Table 3, *p* = 0.02, and *p* = 0.043, respectively). Serum UA was significantly higher in participants with higher android fat content (*p* < 0.001). High sensitivity-CRP, NEFA and leptin plasma levels were not different. Lipid profile indicated higher plasma triglycerides concentration (*p* = 0.006) while total, HDL and LDL cholesterol did not differ. Lipidomic class analysis were performed in a subgroup of subjects (lower android fat: *n* = 21; higher android fat: *n* = 19). Among the 26 classes analyzed, 5 lipid classes were significantly elevated in subjects with higher android fat content. Those were: Ceramide (CER), Diacylglycerol (DG), phosphatidylethanolamine (PE), phosphatidylglycerol (PG) and triacylglycerol (TAG) (Table 3). Among the liver enzymes, ALT was slightly not but significantly higher in subjects with higher android fat content. GGT concentrations were elevated in those with higher android fat content compared to those with lower android fat content (*p* < 0.05).

**TABLE 3:** Biochemical data.

	**Lower android fat content (<9.5% of total fat)**	**Higher android fat content (>9.5% of total fat)**	***P-*value**
Fasting glucose, mmol/L	4.70 ± 0.35	4.64 ± 0.31	0.42
2-h plasma glucose, mmol/L	4.99 ± 1.1	5.96 ± 1.3	0.02
Insulin, mmol/L	17.7 ± 8.3	25.4 ± 11.7	0.043
Total cholesterol, mmol/L	4.47 ± 0.67	4.84 ± 0.89	0.101
HDL cholesterol, mmol/L	1.17 ± 0.22	1.08 ± 0.20	0.297
LDL cholesterol, mmol/L	2.76 ± 0.60	2.89 ± 0.66	0.457
Triglycerides, mmol/L	1.19 ± 0.6	1.9 ± 0.9	0.006
h-CRP (mg/l)^(1)^	0.85 (2.38)	1.50 (1.60)	0.335
ALT (U/L)^(1)^	30 (18)	38 (32)	0.072
GGT (U/L)	27.3 ± 13.7	45.3 ± 28.8	0.017
NEFA,	0.335 ± 0.166	0.414 ± 0.183	0.155
Uric acid, mmol/L	0.376 ± 0.069	0.457 ± 0.076	<0.001
Leptin, ng/mL	21.9 ± 8.7	19.6 ± 12.3	0.568
Lipidomic classes^(2)^			
Total CER, pmol/mL	6.1 ± 1.1	7.4 ± 2.3	0.027
Total DG, pmol/mL	41.4 ± 2.3	79.6 ± 4.7	0.002
Total PE, pmol/mL	20.6 ± 9.6	32.2 ± 1.7	0.012
Total PG, pmol/mL	0.64 ± 0.1	0.94 ± 0.3	0.007
Total TAG, pmol/mL	675 ± 217	994 ± 442	0.006

*HOMA, homeostatic model assessment; CER, ceramide; DG, diacylglycerol, PE, phosphatidylethanolamine; PG, phosphatidylglycerol; TAG, triacylglycerol. Data is reported as mean ± SD or median (interquartile range). ^(1)^Mann–Whitney test, ^(2)^Only lipidomic classes where significant differences were found are reported.*

### Renal Function

The creatinine clearance was assessed using plasma and urine analysis of creatinine and was performed in 20 subjects in each group as two participants failed to return their sample and three others had urine collection <1L. Creatinine clearance was similar: 161 ± 44 ml/min and 159 ± 62 ml/min in the group with low and high android fat content, respectively (*p* = 0.90).

### Digital Vascular Analysis

**FIGURE 1:**
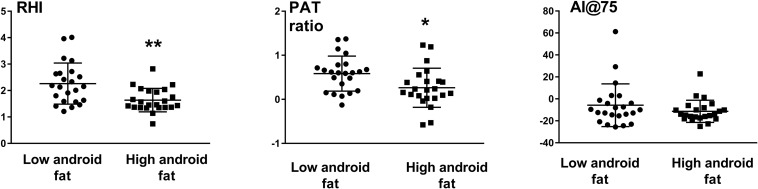
Endothelial function as assessed by the reactive hyperemia index (RHI) and Pulse Amplitude Tonometry (PAT ratio) and augmentation index (AI@75) in subjects with low and high android fat content. ^∗^*P* < 0.05, ^∗∗^*P* < 0.01.

Reactive hyperemia index and pulse amplitude tonometry ratio were significantly less in those with higher android fat content compared to those with lower android fat (RHI: 1.64 ± 0.44 vs. 2.26 ± 0.78, *p* = 0.001 and PAT ratio: 0.26 ± 0.44 vs. 0.58 ± 0.40, *p* = 0.013) (Figure 1). Arterial stiffness as assessed by AI@75 was similar between the two groups (Figure 1).

## Discussion

In this study, we show that for the same level of BMI and fat mass, young overweight males with preferential fat in the android region present with an impaired metabolic profile and endothelial function compared to those with lower android fat content. These differences were observed in the absence of any difference in blood pressure and sympathetic tone.

The group of subjects with higher android fat content presented with reduced insulin sensitivity and decreased glucose tolerance as measured by fasting insulin concentrations and OGTT respectively compared to individuals with lower android fat depot, after correction for BMI. Our study is in line with previous findings demonstrating that excess body fat in abdominal rather than in peripheral fat depot is involved in the development of insulin resistance in adults (Peterson et al., 2015) and children (Aucouturier et al., 2009). This is of particular relevance because decreased insulin sensitivity is thought to be the underlying linkage between obesity, type 2 diabetes and CV disease (Reaven, 2011). Decreased insulin sensitivity in the setting of high android fat depot may reflect structural and functional differences between android and peripheral fat tissue with android tissue possibly expressing higher pro-inflammatory, lipogenic and lipolytic genes and containing higher proportions of saturated fatty acids (Marinou et al., 2014). We found no difference however between the 2 groups in serum CRP and leptin concentrations and, although serum NEFA tended to be higher in the group with higher android fat, it did not reach significance.

Of note in this study was the finding that endothelial function was significantly lower in the group of young males with higher android fat content. Impaired endothelial function is considered an early marker of atherosclerotic disease, with important clinical implications including cardiac dysfunction, coronary artery disease, hypertension, diabetes, and neurologic disorders, leading to increased mortality and morbidity (Kim et al., 2006). Endothelial dysfunction is detectable in overweight children and young adults and develops even after a rapid and modest weight gain of 4 kg (Romero-Corral et al., 2010). Decreased insulin sensitivity observed in the group with high android fat may have important consequences in the development of endothelial dysfunction and atherosclerosis (Muniyappa and Sowers, 2013). The pathway involving decreased endothelial function in this setting of higher android fat remains to be established.

In addition, subjects with higher android fat content were characterized by an abnormal lipid profile in the form of elevated plasma concentration of TG and five other lipidomic classes. Elevated fasting TG levels are a common dyslipidemic feature that accompanies the prediabetic state and is associated with CV risk in young men (Tirosh et al., 2008). Serum TG have previously been reported to be positively associated with android fat in a large study in adults in the general population (Min and Min, 2015). Such abnormal serum TG in those with higher android fat content may negatively impact endothelial function as a strong link between serum TG and endothelial function was demonstrated in a large community-based study (Kajikawa et al., 2016). Among the many lipid classes, some have been implicated in metabolic and CV disease development in animal models and in humans. Within the system-wide lipid network, Stegemann et al. (2014) noted that TAG and PE were most informative for CV disease risk and plasma CER is a significant predictor for CV death beyond currently used lipid markers in patients with coronary artery disease (Laaksonen et al., 2016). While it is uncertain why these lipid species are elevated in those with higher android fat, it may add to their elevated CV risk.

Individuals with higher android fat content were characterized by elevated serum UA compared to those with lower android fat. UA has emerged as an important marker of end organ damage (Lambert et al., 2017a) and CV risk (Borghi et al., 2018). Therefore, increased UA in those with elevated android fat content may be an additional CV risk factor. In line with our findings, a previous study conducted in a large cohort of Chinese subjects indicated that increasing risk of blood pressure outcomes across UA quartiles was most prominent in individuals with abdominal obesity (Yang et al., 2012). Hyperuricemia is strongly associated with an increased risk of atherosclerosis and UA has also been shown to induce vascular endothelial dysfunction via oxidative stress and inflammatory responses (Puddu et al., 2012). However, whether elevated UA in the group of young males with high levels of android fat affects their endothelial function is uncertain because lowering UA fails to improve endothelial function (Borgi et al., 2017).

While low endothelial function was noticed in individuals with higher fat content, we noticed that the arterial stiffness assessed from the augmentation index from the digits as well as the renal function were not different between subjects with higher or lower android fat content. Both arterial stiffness (Corrigan et al., 2017) and decreased kidney function (Oh et al., 2017) have been shown to be affected by fat distribution in older subjects. The young age and absence of cardiometabolic abnormalities in our participants even in the presence of higher android fat may explain the lack of difference. Our results of a lower endothelial function in those with higher android fat depot are different to those of Weil et al. who found that abdominal obesity (assessed with waist circumference) was not associated with greater impairment in endothelial function in overweight and obese adult men (Weil et al., 2011). Discrepancies in the findings may be due to differences in subject age, assessment of endothelial function and assessment of abdominal fat content. Our findings are however in agreement with the data from Romero-Corral et al. (2010) who showed that weight gain induced endothelial dysfunction was significantly linked to visceral but not subcutaneous fat gain.

Overweight is a well-recognized risk factor for pre-hypertension and hypertension and studies have suggested that the risk of developing hypertension may be linked to body fatness and body fat distribution (Wiklund et al., 2008; Ye et al., 2018). Similarly, excess adiposity is characterized by elevated sympathetic nervous system activity, even in young healthy individuals, which is likely to impact on their CV risk including hypertension development (Lambert et al., 2010). Given that MSNA was reported to be 55% higher in men with elevated abdominal visceral fat compared with their age, total fat mass, and abdominal subcutaneous fat-matched peers with lower levels (Alvarez et al., 2002), it seems that sympathetic activation may be an important driver mediating CV risk in those with higher abdominal adiposity. Contrary to expectation, we found that MSNA, expressed as bursts incidence was not different between our subjects with high and low android fat content. Of note burst frequency was significantly higher in participants with higher android fat but this increase was no longer noticed after adjusting for the heart rate. This is surprising considering that sympathetic activation to the skeletal muscle is usually observed in the presence of glucose intolerance (Straznicky et al., 2012) and dyslipidemia (Lambert et al., 2013). Blood pressure and cardiac (vagal) baroreflex function were also found to be similar between the 2 groups suggesting that in this cohort of young overweight males, excess android fat may not further alter hemodynamic control. One exception was noticed for the heart rate which, as noticed above, was higher in those with high android fat content. As the HRV data indicated no differences in cardiac vagal control between the two group, perhaps higher heart rate may reflect preferential sympathetic activation to the heart (Esler et al., 1989).

Limitations of the study include the small number of participants and the cross-sectional aspect of our study which does not permit the determination of causality. The EndoPat technique uses pulse volume changes at the fingertips after an occlusion of the brachial artery as an index of endothelial function. Although the method has been validated (Kuvin et al., 2003) it has a higher within-day variability compared to the more traditional method of flow mediated dilation (Onkelinx et al., 2012). Dietary habits and physical activity were not assessed in these participants hence we are not able to determine if these factors may have influenced our results. Strengths of the study includes the number of different outcomes assessed with regards to both metabolic and end organ damage as well as direct sympathetic nervous system activity measurements and the use of iDXA.

## Conclusion

In conclusion, our study indicated that in young overweight but otherwise healthy males, preferential fat depot in the android region was associated with impaired glucose and lipid profile, increased serum UA concentrations and worsening of endothelial function. On the other hand renal function and arterial stiffness were comparable. Contrary to expectation, sympathetic tone as assessed with MSNA and expressed as burst incidence was not elevated in participants with higher android fat content. These data suggest that elevated android fat may confer heightened CV risk and interventions to slow down the development of CV disease should specifically target android fat.

## Disclosure

MS received research support and speaker fees from Abbott. GH received research support from Boehringer Ingelheim.

## Data Availability

The datasets generated for this study are available on request to the corresponding author.

## Ethics Statement

The studies involving human participants were reviewed and approved by the Alfred Hospital Ethics Committee 14/08 and 168/10. The patients/participants provided their written informed consent to participate in this study.

## Author Contributions

EL, CS, NE, GH, MS, and GL contributed to the conception and design of the study. CS collected the clinical data, organized the database, and performed the statistical analysis. NE and PM performed all the lipidomic analysis. EL and CS wrote the first draft of the manuscript. All authors contributed to the manuscript revision, and read and approved the submitted version.

## Conflict of Interest Statement

The authors declare that the research was conducted in the absence of any commercial or financial relationships that could be construed as a potential conflict of interest.

## References

[B1] Abramowitz M. K. Hall C. B. Amodu A. Sharma D. Androga L. Hawkins M. (2018). Muscle mass, BMI, and mortality among adults in the United States: a population-based cohort study. *PLoS One* 13:e0194697. 10.1371/journal.pone.0194697 29641540 PMC5894968

[B2] Alvarez G. E. Ballard T. P. Beske S. D. Davy K. P. (2004). Subcutaneous obesity is not associated with sympathetic neural activation. *Am. J. Physiol. Heart Circ. Physiol.* 287 H414–H418. 10.1152/ajpheart.01046.2003 14988078

[B3] Alvarez G. E. Beske S. D. Ballard T. P. Davy K. P. (2002). Sympathetic neural activation in visceral obesity. *Circulation* 106 2533–2536. 12427647 10.1161/01.cir.0000041244.79165.25

[B4] Aucouturier J. Meyer M. Thivel D. Taillardat M. Duche P. (2009). Effect of android to gynoid fat ratio on insulin resistance in obese youth. *Arch. Pediatr. Adolesc. Med.* 163 826–831. 10.1001/archpediatrics.2009.148 19736336

[B5] Borghi C. Rodriguez-Artalejo F. De Backer G. Dallongeville J. Medina J. Nuevo J. (2018). Serum uric acid levels are associated with cardiovascular risk score: a post hoc analysis of the EURIKA study. *Int. J. Cardiol.* 253 167–173. 10.1016/j.ijcard.2017.10.045 29306459

[B6] Borgi L. McMullan C. Wohlhueter A. Curhan G. C. Fisher N. D. Forman J. P. (2017). Effect of uric acid-lowering agents on endothelial function: a randomized, double-blind, placebo-controlled trial. *Hypertension* 69 243–248. 10.1161/HYPERTENSIONAHA.116.08488 28028194 PMC5233648

[B7] Calle E. E. Thun M. J. Petrelli J. M. Rodriguez C. Heath C. W. Jr. (1999). Body-mass index and mortality in a prospective cohort of U.S. adults. *N. Engl. J. Med.* 341 1097–1105. 10.1056/NEJM199910073411501 10511607

[B8] Corrigan F. E. III Kelli H. M. Dhindsa D. S. Heinl R. E. Al Mheid I. Hammadah M. (2017). Changes in truncal obesity and fat distribution predict arterial health. *J. Clin. Lipidol.* 11 1354–1360.e3. 10.1016/j.jacl.2017.08.013 28942095 PMC9136712

[B9] Eikelis N. Lambert E. A. Phillips S. Sari C. I. Mundra P. A. Weir J. M. (2017). Muscle sympathetic nerve activity is associated with elements of the plasma lipidomic profile in young Asian adults. *J. Clin. Endocrinol. Metab.* 102 2059–2068. 10.1210/jc.2016-3738 28323975

[B10] Esler M. Jennings G. Lambert G. (1989). Measurement of overall and cardiac norepinephrine release into plasma during cognitive challenge. *Psychoneuroendocrinology* 14 477–481. 2623135 10.1016/0306-4530(89)90047-4

[B11] Guglielmi V. Sbraccia P. (2018). Obesity phenotypes: depot-differences in adipose tissue and their clinical implications. *Eat Weight Disord.* 23 3–14. 10.1007/s40519-017-0467-9 29230714

[B12] Hamburg N. M. Keyes M. J. Larson M. G. Vasan R. S. Schnabel R. Pryde M. M. (2008). Cross-sectional relations of digital vascular function to cardiovascular risk factors in the Framingham heart study. *Circulation* 117 2467–2474. 10.1161/CIRCULATIONAHA.107.748574 18458169 PMC2734141

[B13] Imboden M. T. Welch W. A. Swartz A. M. Montoye A. H. Finch H. W. Harber M. P. (2017). Reference standards for body fat measures using GE dual energy x-ray absorptiometry in caucasian adults. *PLoS One* 12:e0175110. 10.1371/journal.pone.0175110 28388669 PMC5384668

[B14] Indumathy J. Pal G. K. Pal P. Ananthanarayanan P. H. Parija S. C. Balachander J. (2015). Association of sympathovagal imbalance with obesity indices, and abnormal metabolic biomarkers and cardiovascular parameters. *Obes. Res. Clin. Pract.* 9 55–66. 10.1016/j.orcp.2014.01.007 25660176

[B15] Kajikawa M. Maruhashi T. Matsumoto T. Iwamoto Y. Iwamoto A. Oda N. (2016). Relationship between serum triglyceride levels and endothelial function in a large community-based study. *Atherosclerosis* 249 70–75. 10.1016/j.atherosclerosis.2016.03.035 27065244

[B16] Kang S. M. Yoon J. W. Ahn H. Y. Kim S. Y. Lee K. H. Shin H. (2011). Android fat depot is more closely associated with metabolic syndrome than abdominal visceral fat in elderly people. *PLoS One* 6:e27694. 10.1371/journal.pone.0027694 22096613 PMC3214067

[B17] Kim J. A. Montagnani M. Koh K. K. Quon M. J. (2006). Reciprocal relationships between insulin resistance and endothelial dysfunction: molecular and pathophysiological mechanisms. *Circulation* 113 1888–1904. 10.1161/CIRCULATIONAHA.105.563213 16618833

[B18] Kim T. H. Lee S. S. Yoo J. H. Kim S. R. Yoo S. J. Song H. C. (2012). The relationship between the regional abdominal adipose tissue distribution and the serum uric acid levels in people with type 2 diabetes mellitus. *Diabetol. Metab. Syndr.* 4:3. 10.1186/1758-5996-4-3 22301198 PMC3395847

[B19] Kuvin J. T. Patel A. R. Sliney K. A. Pandian N. G. Sheffy J. Schnall R. P. (2003). Assessment of peripheral vascular endothelial function with finger arterial pulse wave amplitude. *Am. Heart J.* 146 168–174. 10.1016/S0002-8703(03)00094-2 12851627

[B20] Laakso M. (1993). How good a marker is insulin level for insulin resistance? *Am. J. Epidemiol.* 137 959–965. 10.1093/oxfordjournals.aje.a116768 8317453

[B21] Laaksonen R. Ekroos K. Sysi-Aho M. Hilvo M. Vihervaara T. Kauhanen D. (2016). Plasma ceramides predict cardiovascular death in patients with stable coronary artery disease and acute coronary syndromes beyond LDL-cholesterol. *Eur. Heart J.* 37 1967–1976. 10.1093/eurheartj/ehw148 27125947 PMC4929378

[B22] Lambert E. Sari C. I. Dawood T. Nguyen J. McGrane M. Eikelis N. (2010). Sympathetic nervous system activity is associated with obesity-induced subclinical organ damage in young adults. *Hypertension* 56 351–358. 10.1161/HYPERTENSIONAHA.110.155663 20625075

[B23] Lambert E. Straznicky N. Sari C. I. Eikelis N. Hering D. Head G. (2013). Dyslipidemia is associated with sympathetic nervous activation and impaired endothelial function in young females. *Am. J. Hypertens.* 26 250–256. 10.1093/ajh/hps016 23382410

[B24] Lambert E. A. Hachem M. Hemmes R. Straznicky N. E. Eikelis N. Sari C. I. (2017a). Serum uric acid and the relationship with subclinical organ damage in adults. *J. Hypertens.* 35 745–752. 10.1097/HJH.0000000000001212 28248904

[B25] Lambert E. A. Sari C. I. Eikelis N. Phillips S. E. Grima M. Straznicky N. E. (2017b). Effects of moxonidine and low-calorie diet: cardiometabolic benefits from combination of both therapies. *Obesity* 25 1894–1902. 10.1002/oby.21962 28865109

[B26] Marinou K. Hodson L. Vasan S. K. Fielding B. A. Banerjee R. Brismar K. (2014). Structural and functional properties of deep abdominal subcutaneous adipose tissue explain its association with insulin resistance and cardiovascular risk in men. *Diabetes Care* 37 821–829. 10.2337/dc13-1353 24186879

[B27] Min K. B. Min J. Y. (2015). Android and gynoid fat percentages and serum lipid levels in United States adults. *Clin. Endocrinol.* 82 377–387. 10.1111/cen.12505 24974911

[B28] Muniyappa R. Sowers J. R. (2013). Role of insulin resistance in endothelial dysfunction. *Rev. Endocr. Metab. Disord.* 14 5–12. 10.1007/s11154-012-9229-1 23306778 PMC3594115

[B29] Oh I. H. Choi J. W. Lee C. H. Park J. S. (2017). Estimating negative effect of abdominal obesity on mildly decreased kidney function using a novel index of body-fat distribution. *J. Korean Med. Sci.* 32 613–620. 10.3346/jkms.2017.32.4.613 28244287 PMC5334159

[B30] Onkelinx S. Cornelissen V. Goetschalckx K. Thomaes T. Verhamme P. Vanhees L. (2012). Reproducibility of different methods to measure the endothelial function. *Vasc. Med.* 17 79–84. 10.1177/1358863X12436708 22402933

[B31] Parati G. Di Rienzo M. Bonsignore M. R. Insalaco G. Marrone O. Castiglioni P. (1997). Autonomic cardiac regulation in obstructive sleep apnea syndrome: evidence from spontaneous baroreflex analysis during sleep. *J. Hypertens.* 15(12 Pt 2), 1621–1626. 9488213 10.1097/00004872-199715120-00063

[B32] Peterson M. D. Al Snih S. Serra-Rexach J. A. Burant C. (2015). Android Adiposity and Lack of Moderate and Vigorous Physical Activity Are Associated With Insulin Resistance and Diabetes in Aging Adults. *J. Gerontol. A Biol. Sci. Med. Sci.* 70 1009–1017. 10.1093/gerona/glv002 25711528 PMC4506315

[B33] Puddu P. Puddu G. M. Cravero E. Vizioli L. Muscari A. (2012). Relationships among hyperuricemia, endothelial dysfunction and cardiovascular disease: molecular mechanisms and clinical implications. *J. Cardiol.* 59 235–242. 10.1016/j.jjcc.2012.01.013 22398104

[B34] Reaven G. M. (2011). Insulin resistance: the link between obesity and cardiovascular disease. *Med. Clin. North Am.* 95 875–892. 10.1016/j.mcna.2011.06.002 21855697

[B35] Romero-Corral A. Sert-Kuniyoshi F. H. Sierra-Johnson J. Orban M. Gami A. Davison D. (2010). Modest visceral fat gain causes endothelial dysfunction in healthy humans. *J. Am. Coll. Cardiol.* 56 662–666. 10.1016/j.jacc.2010.03.063 20705223 PMC3951914

[B36] Rothney M. P. Martin F. P. Xia Y. Beaumont M. Davis C. Ergun D. (2012). Precision of GE lunar iDXA for the measurement of total and regional body composition in nonobese adults. *J. Clin. Densitom.* 15 399–404. 10.1016/j.jocd.2012.02.009 22542222

[B37] Samsell L. Regier M. Walton C. Cottrell L. (2014). Importance of android/gynoid fat ratio in predicting metabolic and cardiovascular disease risk in normal weight as well as overweight and obese children. *J. Obes.* 2014:846578. 10.1155/2014/846578 25302115 PMC4181515

[B38] Snijder M. B. Zimmet P. Z. Visser M. Dekker J. M. Seidell J. C. Shaw J. E. (2004). Independent and opposite associations of waist and hip circumferences with diabetes, hypertension and dyslipidemia: the ausdiab study. *Int. J. Obes. Relat. Metab. Disord.* 28 402–409. 10.1038/sj.ijo.0802567 14724659

[B39] Stegemann C. Pechlaner R. Willeit P. Langley S. R. Mangino M. Mayr U. (2014). Lipidomics profiling and risk of cardiovascular disease in the prospective population-based Bruneck study. *Circulation* 129 1821–1831. 10.1161/CIRCULATIONAHA.113.002500 24622385

[B40] Straznicky N. E. Grima M. T. Sari C. I. Eikelis N. Lambert E. A. Nestel P. J. (2012). Neuroadrenergic dysfunction along the diabetes continuum: a comparative study in obese metabolic syndrome subjects. *Diabetes* 61 2506–2516. 10.2337/db12-0138 22664956 PMC3447913

[B41] Tirosh A. Shai I. Bitzur R. Kochba I. Tekes-Manova D. Israeli E. (2008). Changes in triglyceride levels over time and risk of type 2 diabetes in young men. *Diabetes Care* 31 2032–2037. 10.2337/dc08-0825 18591400 PMC2551650

[B42] Vecchie A. Dallegri F. Carbone F. Bonaventura A. Liberale L. Portincasa P. (2018). Obesity phenotypes and their paradoxical association with cardiovascular diseases. *Eur. J. Intern. Med.* 48 6–17. 10.1016/j.ejim.2017.10.020 29100895

[B43] von Eyben F. E. Mouritsen E. Holm J. Montvilas P. Dimcevski G. Suciu G. (2003). Intra-abdominal obesity and metabolic risk factors: a study of young adults. *Int. J. Obes. Relat. Metab. Disord.* 27 941–949. 10.1038/sj.ijo.0802309 12861235

[B44] Weil B. R. Stauffer B. L. Mestek M. L. DeSouza C. A. (2011). Influence of abdominal obesity on vascular endothelial function in overweight/obese adult men. *Obesity* 19 1742–1746. 10.1038/oby.2011.189 21738236

[B45] Weir J. M. Wong G. Barlow C. K. Greeve M. A. Kowalczyk A. Almasy L. (2013). Plasma lipid profiling in a large population-based cohort. *J. Lipid Res.* 54 2898–2908. 10.1194/jlr.P035808 23868910 PMC3770102

[B46] Wiklund P. Toss F. Weinehall L. Hallmans G. Franks P. W. Nordstrom A. (2008). Abdominal and gynoid fat mass are associated with cardiovascular risk factors in men and women. *J. Clin. Endocrinol. Metab.* 93 4360–4366. 10.1210/jc.2008-0804 18728169

[B47] Yang T. Chu C. H. Bai C. H. You S. L. Chou Y. C. Hwang L. C. (2012). Uric acid concentration as a risk marker for blood pressure progression and incident hypertension: a Chinese cohort study. *Metabolism* 61 1747–1755. 10.1016/j.metabol.2012.05.006 22656272

[B48] Ye S. Zhu C. Wei C. Yang M. Zheng W. Gan D. (2018). Associations of body composition with blood pressure and hypertension. *Obesity* 26 1644–1650. 10.1002/oby.22291 30260578

[B49] Zhang X. Zhu C. Gao J. Mei F. Yin J. Bu L. (2018). Gender difference in the relationship between serum uric acid reduction and improvement in body fat distribution after laparoscopic sleeve gastrectomy in Chinese obese patients: a 6-month follow-up. *Lipids Health Dis.* 17:288. 10.1186/s12944-018-0934-y 30572901 PMC6302487

